# Lumbosacral Discitis and Vertebral Osteomyelitis Caused by ESBL‐Producing *Escherichia coli* in an Immunocompetent Patient

**DOI:** 10.1155/crdi/9438574

**Published:** 2026-06-19

**Authors:** Nicolas Riveros-Neira, Benjamin J. Behers, Palwusha Khan, Patricia Riano-Rivero, Kathryn Lago, Karen M. Hamad

**Affiliations:** ^1^ Internal Medicine Residency, Sarasota Memorial Hospital, Florida State University, Sarasota, Florida, USA, fsu.edu; ^2^ Florida State University College of Medicine, Tallahassee, Florida, USA, fsu.edu; ^3^ Infectious Diseases, Sarasota Memorial Hospital, Florida State University, Sarasota, Florida, USA, fsu.edu

**Keywords:** antimicrobial therapy, ESBL-producing *Escherichia coli*, lumbosacral discitis, multidrug-resistant gram-negative infection, vertebral osteomyelitis

## Abstract

Discitis and vertebral osteomyelitis are rare spinal infections that are difficult to diagnose due to nonspecific presentations. This case report describes a 62‐year‐old immunocompetent female presenting with recurrent lower back pain and imaging findings concerning for lumbosacral discitis. Initial cultures and biopsy were inconclusive, but surgical intervention confirmed vertebral osteomyelitis caused by an extended‐spectrum beta‐lactamase (ESBL)–producing *Escherichia coli*. The patient responded well to targeted carbapenem therapy and was placed on chronic suppressive treatment with minocycline. Our case highlights the diagnostic challenges of lumbosacral discitis and the importance of considering multidrug‐resistant, gram‐negative pathogens even in low‐risk patients.

## 1. Introduction

Discitis is a rare but potentially serious infection of the intervertebral disc space, characterized by inflammation often resulting from bacterial pathogens. Its pathophysiology is primarily hematogenous spread from distant sites, particularly the urinary or gastrointestinal tracts [1, 2]. Alternatively, infection may result from direct inoculation during spinal procedures or extension of infection from adjacent structures [1, 3]. While discitis remains relatively uncommon, epidemiological studies reveal a bimodal age distribution, predominantly affecting children under 10 years and adults over 50, with a higher incidence in older individuals [1, 2]. In developed countries, the estimated annual incidence ranges from 0.4 to 2.4 cases per 100,000, with recent data indicating an increasing trend, particularly among the elderly [1, 2].

*Staphylococcus aureus* remains the most identified causative organism, although *Streptococcus* species and gram‐negative organisms, such as *Escherichia coli* (*E. coli*), have been implicated [1, 2]. Cases of multidrug‐resistant organisms (MDROs) also exist, primarily in healthcare‐associated cases, postoperatively, or in immunocompromised patients [4]. Studies have estimated rates of MDRO vertebral osteomyelitis to be between 15% and 47%, driven mostly by methicillin‐resistant *Staphylococcus aureus* (MRSA) [4, 5]. Of the MDRO cases, only approximately 2% of cases are due to extended‐spectrum beta‐lactamase (ESBL)–producing gram‐negative organisms [4, 5]. We present a case of ESBL *E. coli* discitis in a patient without known risk factors.

## 2. Case Presentation

**FIGURE 1:**
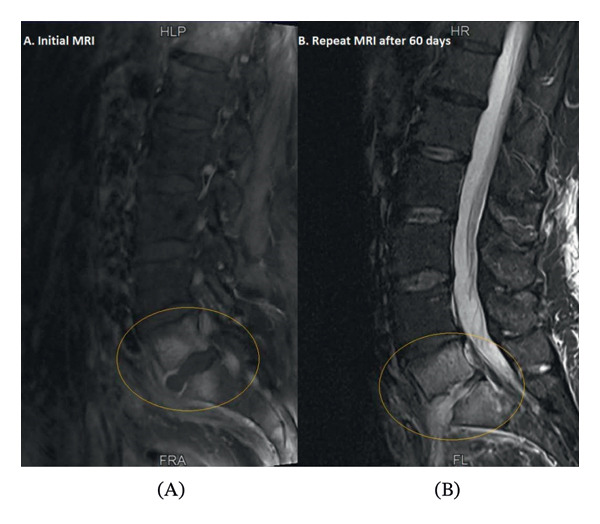
(A) T1 fat‐saturated postcontrast imaging reveals areas of marrow edema, abnormal enhancement of the L5 and S1 endplates, and abnormal enhancement wrapping around the anterior margin of the disc. (B) T2‐weighted STIR TSE reveals an abnormal T2 hyperintense signal within the disc, which heterogeneously enhances with diffuse enhancement of the annulus fibrosis and extension of inflammatory phlegmonous change into the retroperitoneal anterior paravertebral soft tissues.

A 62‐year‐old female was referred to our institution by her primary care physician after an outpatient magnetic resonance imaging (MRI) of the lumbar spine for progressive lower back pain showed a possible epidural abscess. Her past medical history was notable for prediabetes, recurrent bacterial vaginosis, and cystocele/rectocele/enterocele status postrobotic‐assisted laparoscopic sacrocolpopexy and obturator tape placement via cystourethroscopy 7 months prior. In addition, 2 months prior, she was hospitalized with 1 week of lower back pain. MRI lumbar spine and sacrum with contrast at that time was suggestive of possible discitis/osteomyelitis at the L5‐S1 disc space. Interventional radiology performed a biopsy, and cultures were inconclusive, so osteomyelitis could not be ruled out. Therefore, intravenous (IV) daptomycin and cefepime were initiated empirically for 6 weeks. She followed up as an outpatient with infectious disease (ID), reporting resolution of her back pain. Two weeks after completion of antibiotics, she presented to her primary care physician with recurrence of lower back pain, leading to the outpatient MRI concerning for disease progression compared to prior studies (Figure 1).

**FIGURE 2:**
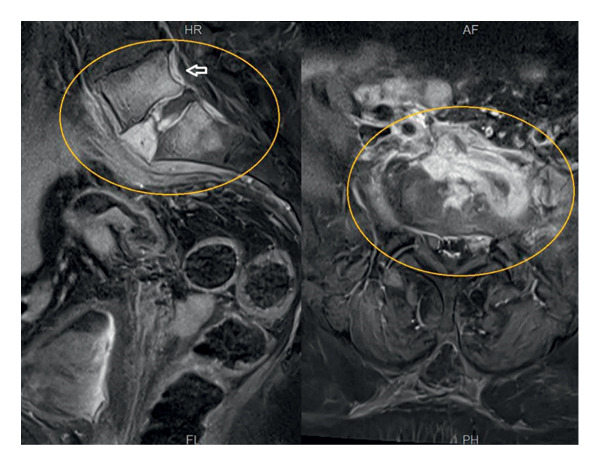
T1 TSE fat‐suppression revealed low T1 signal within the vertebral bodies, with extensive edema/infection that appears to extend into the left sacrum and left anterior sacral ala and a possible 1.9 × 0.4 cm epidural abscess posterior to L5‐S1.

On presentation, she was afebrile and hemodynamically stable with blood pressure and pulse within normal parameters. Physical exam was notable for moderate pain to palpation over the lumbar spinal process and mildly decreased strength in the right lower extremity, 3/5, but no signs of spinal cord compression. Laboratory values included a normal white blood cell count of 6.7 × 10^3^ cells/μL (normal: 4.5–11.0 10^3^ cells/μL) with normal differential, mildly elevated C‐reactive protein of 1.1 mg/dL (normal < 0.3 mg/dL), and elevated erythrocyte sedimentation rate of 57 mm/hr (normal: 0–30 mm/hr). Urine and blood cultures were negative. MRI lumbar spine and sacrum with contrast was consistent with L5‐S1 osteomyelitis and possible epidural abscess posterior to L5‐S1 (Figure 2). Given the concern for epidural abscess with significant back pain, empiric antibiotic therapy was initiated with IV vancomycin and IV cefepime due to the absence of culture data from prior biopsy.

**FIGURE 3:**
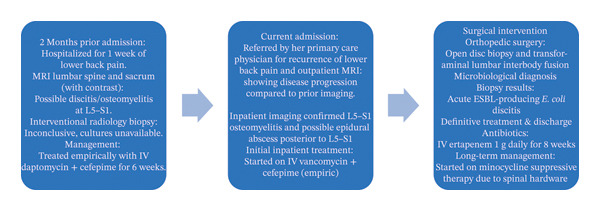
Timeline of our case.

Orthopedic surgery performed resection and debridement of the infected L5‐S1 disc space with open‐disc biopsy and transforaminal lumbar interbody fusion with cage placement. Biopsy confirmed the diagnosis of acute discitis, and cultures grew ESBL *E. coli*, susceptible to ertapenem (Table 1). After 6 days of hospitalization, she was discharged on IV ertapenem 1 g daily for 8 weeks with weekly follow‐up with ID. Upon completion of antibiotics, she was started on minocycline suppression therapy given the presence of spinal hardware. A timeline of this case can be seen in Figure 3.

**TABLE 1:** Cultures from open‐disc biopsy grew ESBL *E. coli*.

Antibiotic	Susceptibility
Ampicillin	Resistant > 16
Ampicillin/sulbactam	Susceptible 8/4
Cefepime	Resistant 4
Ceftriaxone	Resistant > 32
Ciprofloxacin	Resistant > 2
Ertapenem	Susceptible ≤ 0.25
Gentamicin	Susceptible ≤ 2
Meropenem	Susceptible ≤ 0.5
Piperacillin/tazobactam	Susceptible ≤ 2/4
Tetracycline	Resistant > 8
Tobramycin	Susceptible ≤ 2
Trimethoprim/sulfamethoxazole	Resistant > 2/38
Minocycline	Susceptible 2

*Note:* Numbers next to susceptibilities are minimal inhibitory concentration (MIC) calculated using the BD Phoenix panel.

## 3. Discussion

Our case highlights a rare presentation of lumbosacral discitis with subsequent progression to vertebral osteomyelitis caused by an ESBL‐producing *E*. *coli* in a patient without risk factors. Gram‐negative vertebral osteomyelitis remains an uncommon form of spinal infection [6]. In addition, sacral involvement in vertebral osteomyelitis is rare, with an incidence of only 0.1%, while the lumbar spine (60.3%) and thoracic spine (31.6%) are more frequently affected regions [6, 7]. Therefore, the presence of both of these factors occurring simultaneously highlights the rarity of our case.

Community‐acquired infections due to ESBL‐producing *E*. *coli* are increasing significantly, representing a worrisome therapeutic challenge due to limitations in effective antibiotic options [8]. A study analyzing 213 gram‐negative isolates from various clinical samples (both inpatient and outpatient) showing resistance to third‐generation cephalosporins found that 69% were ESBL producers [8]. Furthermore, *E*. *coli* was the most common isolate and exhibited ESBL production in 81% of its isolates, while also demonstrating multidrug resistance to commonly used antibiotics, such as fluoroquinolones, aminoglycosides, and β‐lactam/β‐lactamase inhibitor combinations [8]. This study encapsulates the spread of ESBL organisms beyond hospital settings and into the community, reducing the reliability of standard empirical therapies and raising concerns about further antibiotic resistance. It also highlights the importance of following culture data to ensure appropriate antibiotics are utilized.

Spinal epidural abscesses (SEA) have increased in incidence in recent decades, largely due to an aging population, higher prevalence of comorbidities, and greater exposure to risk factors, such as IV drug use and spinal procedures [9]. Despite improved imaging and awareness, diagnosis remains difficult because of nonspecific presentations, often leading to delays that worsen outcomes [9]. Prognosis is strongly dependent on early recognition and neurologic status at presentation, with delayed treatment associated with irreversible deficits, including paralysis [9]. Although mortality has declined to 1%–16% of cases, this remains significant, particularly in high‐risk patients [9]. Surgical decompression is the standard of care, but is associated with considerable operative risk, especially in older populations with numerous comorbidities [9]. Furthermore, failure of nonoperative management occurs in up to 41% of cases and is associated with poor neurologic recovery, highlighting the importance of timely diagnosis and appropriate management [9].

Another notable aspect of our case is that the patient lacked traditional risk factors for vertebral discitis/osteomyelitis. These include advanced age, diabetes mellitus, immunosuppressive states, structural spinal abnormalities, recent systemic or localized infections, IV drug use, and a history of spinal trauma [7, 10]. Although she had no traditional risk factors, she did have a urinary tract infection (UTI) approximately 2 years prior, and culture grew ESBL *E. coli* with similar sensitivities to the microorganism isolated on disc biopsy surgical cultures (Table 2). Prior studies have shown an association between UTIs and the development of vertebral osteomyelitis, with rates of the latter occurring an estimated 15%–37% of the time [11–13]. In addition, our patient’s history of recurrent bacterial vaginosis may have also played a role. Studies have shown bacterial vaginosis to be associated with an increased risk of UTI in both pregnant and nonpregnant women, with odds ratios of 2.21 and 2.79, respectively [14–16]. Furthermore, studies have shown increased antimicrobial resistance genes in the vaginal microbiome of women with symptomatic bacterial vaginosis, as well as biofilm formation, further contributing to antibiotic resistance and treatment failure [17, 18]. However, the direct relationship with MDROs, particularly ESBL producers, remains unclear.

**TABLE 2:** Cultures showing ESBL *E. coli* with antibiotic susceptibilities from urine culture 2 years prior and disc biopsy.

Antibiotic	ESBL *E. coli* urine culture (two years prior)	ESBL *E. coli* disc biopsy culture (current)
Ampicillin	Resistant > 16	Resistant > 16
Ampicillin/sulbactam	Susceptible 8/4	Susceptible 8/4
Cefepime	Resistant 4	Resistant 4
Ceftriaxone	Resistant > 32	Resistant > 32
Ciprofloxacin	Resistant > 2	Resistant > 2
Ertapenem	Susceptible ≤ 0.25	Susceptible ≤ 0.25
Gentamicin	Susceptible ≤ 2	Susceptible ≤ 2
Meropenem	Susceptible ≤ 0.5	Susceptible ≤ 0.5
Piperacillin/tazobactam	Susceptible 4/4	Susceptible ≤ 2/4
Tetracycline	Resistant > 8	Resistant > 8
Tobramycin	Susceptible ≤ 2	Susceptible ≤ 2
Trimethoprim/sulfamethoxazole	Resistant > 2/38	Resistant > 2/38
Minocycline	Not analyzed	Susceptible 2

*Note:* Numbers next to susceptibilities are minimal inhibitory concentration (MIC) calculated using the BD Phoenix panel.

Vertebral osteomyelitis has a pathophysiology driven primarily by either hematogenous dissemination or contiguous spread from adjacent infected tissues [19, 20]. Hematogenous dissemination via the Batson venous plexus, a valveless venous network that facilitates retrograde flow between the pelvic organs and the vertebral column, has also been suggested [19, 20]. This etiology typically manifests in patients with diabetes, chronic kidney disease, immunosuppression, indwelling vascular catheters, or recent invasive procedures [19, 20]. Although our patient lacked these typical characteristics, we hypothesize dissemination via the Batson venous plexus is what occurred in our case, given urine culture 2 years prior showed ESBL *E. coli* with remarkably similar susceptibility patterns. In addition, blood and urine cultures during this hospitalization were negative, further suggesting the prior UTI as the source. Furthermore, vertebral osteomyelitis can take months to develop, helping to explain the long latency period observed between the UTI and discitis/osteomyelitis in our case.

Pathogen identification through blood and surgical cultures is a critical component in the management of vertebral osteomyelitis to establish targeted antimicrobial therapy [10]. Approximately 30%–60% of vertebral osteomyelitis cases are diagnosed solely through blood cultures, despite studies suggesting this method can miss up to 20% of cases caused by gram‐positive cocci and 50% caused by gram‐negative rods [21, 22]. Biopsy is performed when blood cultures are negative or when polymicrobial or atypical infection is suspected [23]. Although the overall proportion of cases diagnosed by biopsy varies, large cohort studies suggest about 40%–70% of microbiologically confirmed cases require biopsy for diagnosis [21, 22]. However, studies have also shown disc biopsies to have a diagnostic yield as low as 43.4%, while yields can be increased by up to 13% with repeat biopsy, contingent upon image‐guidance accuracy, specimen quality, biopsy technique, and timing of the procedure [24, 25]. Notably, in our case, identification of the causative organism was challenging due to inconclusive results from the initial disc biopsy and negative blood cultures. These inconclusive results on initial biopsy also led to empiric treatment with daptomycin and cefepime, neither of which was ultimately susceptible to the identified organism on repeat biopsy.

Conservative management with antibiotic therapy is effective in most cases of vertebral osteomyelitis [23]. Antibiotic selection should be based on culture susceptibility results, utilizing agents with adequate tissue penetration for a 6‐week course in most cases [23]. Once culture data and sensitivities were available, our patient was switched to ertapenem to allow for once daily dosing and antibiotic administration with home health. Ertapenem is recognized as an excellent option for outpatient IV antibiotics in patients with spinal infections due to its favorable distribution into bone and synovial tissues, as well as once daily dosing [26, 27]. However, ertapenem treatment failures have been reported with once daily dosing for various infections, prompting further investigation into its optimal dosing [27]. In fact, one study on the pharmacokinetics/pharmacodynamics of ertapenem for bone and joint infections recommends the use of twice daily dosing for pathogens with a MIC of > 1 mg/L [27]. Given the MIC in our case was ≤ 0.25 and the improvement in our patient’s symptoms, we feel that she was adequately treated with the once daily dosing. These prior studies highlight the importance of assessing for ertapenem treatment failure with the use of once daily dosing.

Despite generally favorable outcomes, treatment failure occurs in 10%–30% of cases treated conservatively [23]. Surgical intervention is reserved for cases with worsening neurological symptoms, spinal instability, or infection unresponsive to medical therapy [7]. In fact, spinal instability is responsible for surgical intervention in approximately 10%–15% of vertebral osteomyelitis cases [28]. Cases of MDRO vertebral osteomyelitis carry a treatment failure rate of approximately 20%–30%, while also being associated with an increased risk of relapse and need for surgical intervention [23, 29]. In our case, surgical intervention with transforaminal lumbar interbody fusion was necessary due to the significant extension of the infection.

Due to the severity of infection and placement of an interbody fusion cage during surgery, our patient was also started on chronic suppression therapy with minocycline to prevent recurrence. Evidence for using minocycline as chronic suppressive therapy is limited and mostly indirect. Data from prosthetic joint infections suggest tetracyclines, including minocycline, are reasonably effective for suppression therapy and generally well‐tolerated, although these findings do not directly apply to vertebral hardware infections [30]. In addition, a case report documenting the successful treatment of *Cutibacterium modestum* vertebral osteomyelitis with minocycline supports its tissue penetration and efficacy potential for suppressive therapy [31]. However, long‐term use of minocycline has been associated with an increased risk of systemic lupus erythematosus, autoimmune hepatitis, and polyarteritis nodosa, generally occurring after 1 year of use [32]. In addition, antibiotic use increases the risk of *Clostridioides difficile* infection, and prior studies have shown rates ranging from 2% to 22% amongst patients on chronic antibiotic suppression therapy [33]. A thorough risk‐benefit analysis and discussion with the patient should take place prior to initiation of chronic suppression therapy. In addition, clinicians should monitor for side effects in patients on chronic suppression therapy with prompt discontinuation of the drug upon recognition.

## 4. Conclusion

Vertebral osteomyelitis poses significant diagnostic and therapeutic challenges, particularly in patients without classic risk factors or typical clinical presentations. Gram‐negative organisms are a rare cause of vertebral osteomyelitis/discitis. Identification of the causative organism with a 6‐week course of targeted antibiotic therapy is the recommended treatment course, although surgical intervention is indicated in specific instances. Chronic suppression therapy for prevention of recurrent infections can be considered, but should accompany a thorough risk‐benefit analysis, given complications of long‐term antibiotic use.

## Author Contributions

Nicolas Riveros‐Neira, Benjamin J. Behers, and Palwusha Khan: conceptualization, investigation, writing, and editing. Patricia Riano‐Rivero: review, editing, and investigation. Karen M. Hamad and Kathryn Lago: supervision, review, and editing.

## Funding

No specific funding was received.

## Ethics Statement

The authors have nothing to report.

The patient provided written informed consent to publish this case report and accompanying images. The case was approved by an IRB Ethical Approval.

## Consent

Please see the Ethics Statement.

## Conflicts of Interest

The authors declare no conflicts of interest.

## Data Availability

Data sharing is not applicable to this article as no datasets were generated or analyzed during the current study.
